# p47Phox/CDK5/DRP1-Mediated Mitochondrial Fission Evokes PV Cell Degeneration in the Rat Dentate Gyrus Following Status Epilepticus

**DOI:** 10.3389/fncel.2017.00267

**Published:** 2017-09-01

**Authors:** Ji-Eun Kim, Tae-Cheon Kang

**Affiliations:** Department of Anatomy and Neurobiology, Institute of Epilepsy Research, College of Medicine, Hallym University Chuncheon, South Korea

**Keywords:** apocynin, epilepsy, mitochondria, mitochondrial fragmentation, roscovitine, TUNEL

## Abstract

Parvalbumin (PV) is one of the calcium-binding proteins, which plays an important role in the responsiveness of inhibitory neurons to an adaptation to repetitive spikes. Furthermore, PV neurons are highly vulnerable to status epilepticus (SE, prolonged seizure activity), although the underlining mechanism remains to be clarified. In the present study, we found that p47Phox expression was transiently and selectively increased in PV neurons 6 h after SE. This up-regulated p47Phox expression was accompanied by excessive mitochondrial fission. In this time point, CDK5-tyrosine 15 and dynamin-related protein 1 (DRP1)-serine 616 phosphorylations were also increased in PV cells. Apocynin (a p47Phox inhibitor) effectively mitigated PV cell loss via inhibition of CDK5/DRP1 phosphorylations and mitochondrial fragmentation induced by SE. Roscovitine (a CDK5 inhibitor) and Mdivi-1 (a DRP1 inhibitor) attenuated SE-induced PV cell loss by inhibiting aberrant mitochondrial fission. These findings suggest that p47Phox/CDK5/DRP1 may be one of the important upstream signaling pathways in PV cell degeneration induced by SE via excessive mitochondrial fragmentation.

## Introduction

Temporal lobe epilepsy (TLE) is the progressive development of spontaneous recurrent seizures pathologically characterized by specific morphological and cellular alterations (Kang et al., 2006; Kim et al., 2008, 2011). The neuronal death has been a focus of interest in TLE research since specific patterns of neuron loss occur both in principal neurons and in interneurons (Kang et al., 2006; Soukupová et al., 2014). Parvalbumin (PV) is one of the calcium-binding proteins, which are spatial and temporal control of calcium transients across membranes and inside the cytoplasm (Celio, 1990). PV expresses in γ- aminobutyric acid (GABA)-ergic interneurons, mainly in basket cells and axoaxonic cells (Celio, 1990). Since PV regulates the fast-spiking capability of the GABAergic neurons, it plays an important role in the responsiveness of inhibitory neurons to an adaptation to repetitive spikes (Cammarota et al., 2013; Elgueta et al., 2015). Furthermore, PV neurons are extremely vulnerable to status epilepticus (SE, prolonged seizure activity). Indeed, PV neurons are very rapidly degenerated in the hilus of the dentate gyrus 1 day after SE (Soukupová et al., 2014). Therefore, PV neuron loss allows the development of uncontrolled discharges in the epileptic hippocampus. However, the underlining mechanisms for a selective PV cell loss induced by SE remain to be clarified.

Oxidative stress by reactive oxygen species (ROS) appears to be one of the factors contributing to the pathogenesis of neuronal damages in TLE. ROS is generated during normal cellular respiration/metabolic process as well as by specific enzymes such as NADPH oxidase (Nox). Nox is a multi-component enzyme and is composed of the three cytosolic proteins, p40Phox, p47Phox, and p67Phox, and two membrane proteins, which include gp91Phox and p22Phox. In resting stage, Nox components are spatially separated and the complex inactive. In active stage, Nox components form complex, translocate to the membrane, and synthesize ROS (Bedard and Krause, 2007; Brandes et al., 2014). Although apocynin (a p47Phox inhibitor; ’t Hart et al., 1990; Stolk et al., 1994) exhibits neuroprotection in pilocarpine-induced SE models (Pestana et al., 2010; Di Maio et al., 2011) and methamphetamine (MA)-mediated neuronal toxicity (Dang et al. (2016), the role of Nox-mediated ROS synthesis in the selective vulnerability of PV neurons are not completely understood. Therefore, the aims of this study were: (1) to identify whether enhanced Nox activity practically involves a selective PV neuronal loss following SE; and (2) to elucidate the molecular and cellular events responsible for Nox-mediated PV neuron degeneration. Here, we demonstrate that SE transiently up-regulated p47Phox expression in PV neurons, which promoted cyclin-dependent kinase 5 (CDK5) activation. In turn, CDK5 activation degenerated PV neurons by excessive dynamin-related protein 1 (DRP1)-mediated mitochondrial fission. Therefore, these findings suggest that p47Phox/CDK5/DRP1-mediated mitochondrial fission may play an important role in loss of PV neurons following SE.

## Materials and Methods

### Experimental Animals and Chemicals

Male Sprague–Dawley (SD) rats (7 weeks old) were kept under controlled environmental conditions (23 – 25°C, 12 h light/dark cycle) with free access to water and standard laboratory food. All animal protocols were approved by the Administrative Panel on Laboratory Animal Care of Hallym University that comply with NIH Guide for the Care and Use of Laboratory Animals. All possible efforts were taken to avoid animals’ suffering and to minimize the number of animals used during the experiment. All reagents were obtained from Sigma–Aldrich (St. Louis, MO, United States), except as noted.

### Intracerebroventricular Infusion

Under Isoflurane anesthesia (3% induction, 1.5 – 2% for surgery and 1.5% maintenance in a 65:35 mixture of N_2_O:O_2_), animals were stereotaxically implanted a brain infusion kit 1 (Alzet, Cupertino, CA, United States) into the right lateral ventricle (1 mm posterior; 1.5 mm lateral; -3.5 mm depth to the bregma). The infusion kit was sealed with dental cement and connected to an osmotic pump (1007D, Alzet, Cupertino, CA, United States) containing (1) vehicle, (2) apocynin (150 μM), (3) roscovitine (100 μM) or (4) Mdivi-1 (50 μM). In a pilot study and our previous study (Hyun et al., 2017), the concentration of each compound could not affect the seizure latency and its severity in response to pilocarpine. Osmotic pump was placed in a subcutaneous pocket in the interscapular region.

### SE Induction

Three days after surgery, LiCl (3 mEq/kg, i.p.) was administrated to all rats 24 h before an injection of pilocarpine (pilocarpine hydrochloride, 30 mg/kg, i.p.) in the experimental groups or saline (vehicle) in the control group. All animals were given a subcutaneous injection of atropine methylbromide (5 mg/kg, i.p.) 20 min before pilocarpine or saline (control). Two hours after SE onset, diazepam (Valium; Hoffman la Roche, Neuilly sur-Seine, France; 10 mg/kg, i.p.) was administered and repeated, as needed.

### Tissue Processing

At the designated time points (control, 6 h and 12 h after SE induction), rats were perfused transcardially first with phosphate-buffered saline (PBS) followed by a fixative solution (4% paraformaldehyde in 0.1 M phosphate buffer, pH 7.4) during 30 min under urethane anesthesia (1.5 g/kg, i.p.). The brains were removed and submerged in the same fixative solution for 4 h at 4°C. Following postfixation, brains were cryoprotected overnight in 30% sucrose solution (in 0.1 M PBS), and sectioned with a cryostat at 30 μm, and consecutive sections were contained in six-well plates containing PBS.

### Immunohistochemistry and TUNEL Staining

Free-floating sections were first incubated with 10% normal goat serum (Vector, Burlingame, CA, United States) in PBS for 30 min at room temperature. Sections were then incubated in the mixture of primary antibodies in **Table 1** (in PBS containing 0.3% triton X-100) at room temperature for overnight. After washing in PBS, sections were incubated for 1 h in a FITC- or Cy3-conjugated secondary antiserum (Kim and Kang, 2015). To analyze the neuronal damage, we also performed TUNEL staining with the TUNEL apoptosis detection kit (Merck Millipore, Billerica, MA, United States) according to the manufacturer’s instructions, before p47Phox immunofluorescence staining. For nuclei counterstaining, Vectashield mounting medium with DAPI (Vector, Burlingame, CA, United States) was used as a mountant. Some sections (reacted with p47Phox antibody and PV antibody) were reacted with biotinylated secondary antiserum and ABC complex. Thereafter, immunoreactivity was developed by standard DAB reaction. The antibody that was preincubated with pre-immune serum was used as for negative control. As the result of negative control test, no immunoreactive structure was observed (data not shown). Images were captured using an AxioImage M2 microscope or a confocal laser scanning microscope (LSM 710, Carl Zeiss Inc., Oberkochen, Germany).

**Table 1:** Primary antibodies used in the present study.

Antigen	Host	Manufacturer	Dilution
		(catalog number)	used
β-actin	Mouse	Sigma (A5316)	1:6000 (WB)
Mitochondrial marker (Mitochondrial complex IV subunit 1, MTCO1)	Mouse	Abcam (#ab14705)	1:500 (IF)
*N*-Cadherin	Rabbit	Abcam (#ab18203)	1:1000 (WB)
p47Phox	Rabbit	ABBIOTEC (#252159)	1:200 (IF) 1:500 (WB)
pCDK5-Y15	Rabbit	GeneTex (#GTX 32375)	1:100 (IF)
pDRP1-S616	Rabbit	Cell Signaling (#4494)	1:500 (IF)
PV	Mouse	Millipore (#MAB1572)	1:1000 (IF)
PV	Goat	Swant (#PVG213)	1:100000 (IF)

### Subcellular Fraction and Western Blots

To analyze subcellular localization of p47Phox, we used subcellular Protein Fractionation Kit for Tissues (Thermo scientific, Waltham, MA, United States), according to the manufacturer’s instructions. Thereafter, the protein concentration in the supernatant was determined using a Micro BCA Protein Assay Kit (Pierce Chemical, Rockford, IL, United States). Western blotting was performed according to standard procedures. Briefly, Tissue lysate proteins were blotted onto nitrocellulose transfer membranes, then incubated with rabbit-anti p47Phox as a primary antibody (**Table 1**). Immunoreactive bands were detected and quantified on ImageQuant LAS4000 system (GE Healthcare, Piscataway, NJ, United States). The rabbit anti-β-actin primary antibody (for cytosolic fraction) or rabbit anti-N cadherin (for membrane fraction) was used as internal reference (**Table 1**).

### Cell Count and Measurement of Mitochondrial Length

Images of the dentate gyrus were captured (15 sections per each animal), and areas of interest (1 × 10^5^ μm^2^) were selected Thereafter, immunoreactive neurons were counted on 20× images using AxioVision Rel. 4.8 Software. Individual mitochondrion length in PV cells (*n* = 20/section) was measured by using ZEN lite software (Blue Edition, Carl Zeiss Inc., Oberkocken, Germany) following 3D-reconstruction: Based on our previous study (Ko et al., 2016), twenty five serial images (z-stack, 1 μm) were obtained from each hippocampal section. Serial images were stacked, alighned, visualized and converted to 3D images using ZEN lite program. Thereafter, individual mitochondrial length (long axis) was measured. Two different investigators who were blind to the classification of tissues performed cell counts and measurement of mitochondrial length.

### Quantification of Data and Statistical Analysis

All data were analyzed using Mann–Whitney test or ANOVA to determine statistical significance. Bonferroni’s test was used for *post hoc* comparisons. A *p*-value below 0.05 was considered statistically significant.

## Results

### p47Phox-Mediated PV Cell Loss Following SE

**FIGURE 1:**
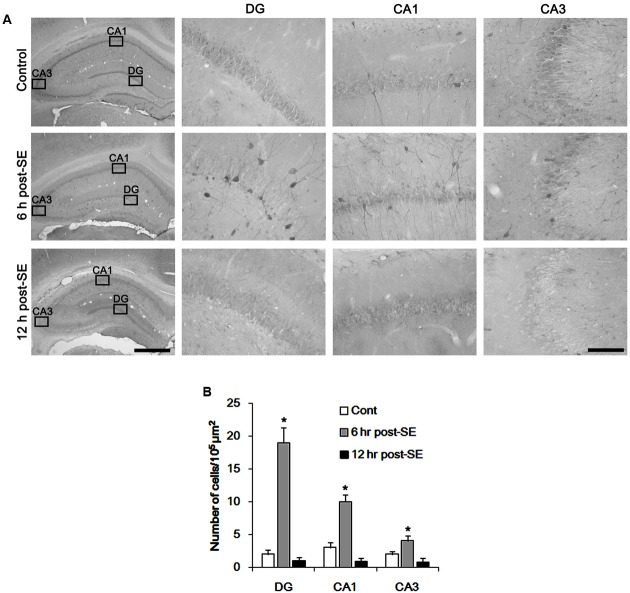
Transient up-regulation of p47Phox in hilar neurons following SE. **(A)** p47Phox expression is rapidly increased in hilar neurons 6 h after SE, and disappears 12 h after SE. Rectangles in low magnification panels indicate the zoom areas for the high magnification photos. Bar = 400 (Left panel) and 50 μm. **(B)** Quantification of the number of p47Phox-positive neurons. Open circles indicate each individual value. Horizontal bars indicate mean value. Error bars indicate SD (*^∗^p* < 0.05 vs. control; *n* = 7, respectively).

**FIGURE 2:**
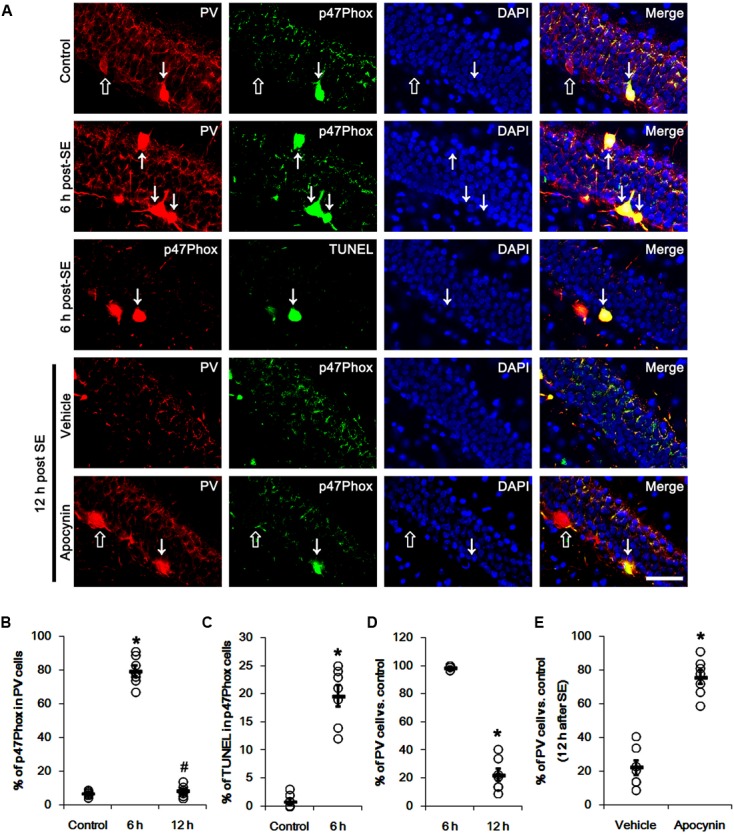
Effect of apocynin on p47Phox expression and PV cell loss following SE. **(A)** Representative photos demonstrating p47Phox expression and TUNEL signal in PV cells. As compared to control, up-regulated p47Phox expression and TUNEL signal are observed in PV cells 6 h after SE. Apocynin attenuates SE-induced PV cell loss 12 h after SE. Arrows indicate PV cells showing up-regulated p47Phox expression and TUNEL signal. Open arrows indicate PV cells showing the absence of p47Phox expression. Bar = 25 μm. **(B)** Quantification of the fraction of p47Phox positive cells in PV neurons. Open circles indicate each individual value. Horizontal bars indicate mean value. Error bars indicate SEM (*^∗^,^#^p* < 0.05 vs. control and 6 h post SE-group, respectively; *n* = 7, respectively). **(C)** Quantification of the fraction of TUNEL positive cells in PV neurons. Open circles indicate each individual value. Horizontal bars indicate mean value. Error bars indicate SEM (*^∗^p* < 0.05 vs. control; *n* = 7, respectively). **(D)** Quantification of the number of PV neurons following SE. Open circles indicate each individual value. Horizontal bars indicate mean value. Error bars indicate SEM (*^∗^p* < 0.05 vs. control; *n* = 7, respectively). **(E)** Quantification of the effect of apocynin on SE-induced PV cell loss. Open circles indicate each individual value. Horizontal bars indicate mean value. Error bars indicate SEM (*^∗^p* < 0.05 vs. vehicle; *n* = 7, respectively).

**FIGURE 3:**
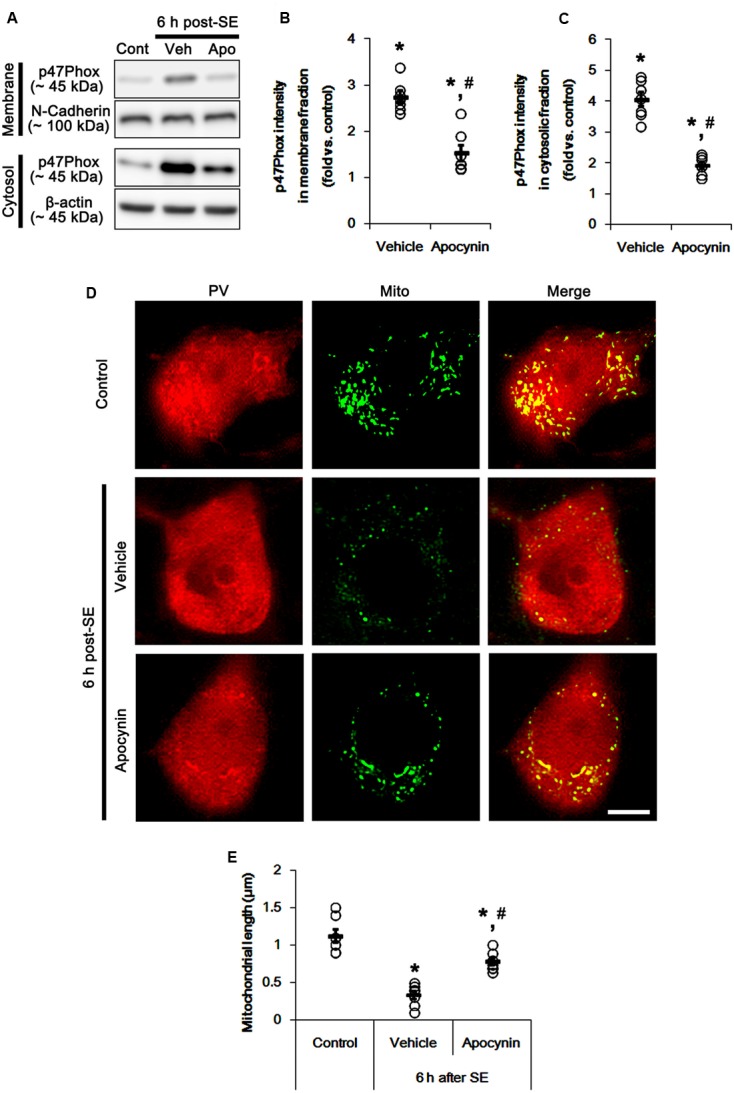
Effect of apocynin on p47Phox translocation and mitochondrial fission induced by SE. **(A)** Representative western data demonstrating p47Phox subcellular locations. As compared to control animals, SE increases p47Phox expression and its membrane location. Apocynin inhibits up-regulation and membrane translocation of p47Phox following SE. **(B)** Quantification of the effect of apocynin on membrane p47Phox translocation following SE. Open circles indicate each individual value. Horizontal bars indicate mean value. Error bars indicate SEM (*^∗^,^#^p* < 0.05 vs. control and vehicle, respectively; *n* = 7, respectively). **(C)** Quantification of the effect of apocynin on cytosolic p47Phox expression following SE. Open circles indicate each individual value. Horizontal bars indicate mean value. Error bars indicate SEM (*^∗^,^#^p* < 0.05 vs. control and vehicle, respectively; *n* = 7, respectively). **(D)** Representative photos of mitochondria in PV cells. As compared to vehicle, apocynin attenuates SE-induced mitochondrial fragmentation in PV cells. Bar = 5 μm. **(E)** Quantification of the effect of apocynin on mitochondrial length. Open circles indicate each individual value. Horizontal bars indicate mean value. Error bars indicate SEM (*^∗^,^#^p* < 0.05 vs. control and vehicle, respectively; *n* = 7, respectively).

**Figure 1** shows that p47Phox expression was selectively increased in hilar neurons 6 h after SE, as compared to control animals (*p* < 0.05 vs. control animals, **Figures 1A,B**). This up-regulated p47Phox expression disappeared in hilar neurons 12 h after SE (*p* < 0.05 vs. control animals, **Figures 1A,B**). Double immunofluorescent study revealed that 80% of PV cells contained p47Phox expression 6 h after SE (**Figures 2A,B**). In addition, 20% of p47Phox positive neurons showed TUNEL positivity at this time point (**Figures 2A,C**), although there was no difference in the number of PV cell as compared to control (**Figures 2A,D**). Twelve hour after SE, the number of PV cells was significantly reduced, as compared to control (**Figures 2A,D**). To elucidate the role of p47Phox in SE-induced PV cell loss, we applied apocynin prior to SE induction. As compared to vehicle, apocynin attenuated SE-induced PV cell loss (**Figures 2A,E**). These findings indicate that disappearance of p47Phox cell may be due to PV cell loss, and that transient up-regulated p47Phox may play an important role in SE-induced PV cell loss. Furthermore, apocynin inhibited up-regulation and membrane translocation of p47Phox in PV cells following SE (*p* < 0.05 vs. vehicle, **Figures 3A–C** and Supplementary Figure 1). Since Nox activation is required for the mobilization of cytoplasmic p47Phox to dock with the membrane-bound proteins for superoxide production (Brandes et al., 2014), our findings indicate that up-regulated p47Phox may be an indicative of increased Nox activity, which is abrogated by apocynin.

### p47Phox-Mediated Mitochondrial Fragmentation in PV Cell Following SE

Mitochondria are dynamic organelles that are essential for maintaining cell function and survival. Thus, impaired mitochondrial dynamics (fission and fusion) triggers neuronal death (Youle and Karbowski, 2005; Parone et al., 2008; DuBoff et al., 2012; Kim et al., 2014). Thus, we investigated whether p47Phox over-expression involves aberrant mitochondrial dynamics in PV cell following SE. In control animals, mitochondrial length was ∼1.13 μm in PV cells (**Figures 3D,E**). Six h after SE, mitochondrial length in PV cells was reduced to ∼0.34 μm (*p* < 0.05 vs. control, **Figures 3D,E**). Apocynin prevented mitochondrial fragmentation induced by SE, thus mitochondrial length was ∼0.78 μm (*p* < 0.05 vs. vehicle, **Figures 3D,E**). These findings indicate a potential relationship between p47Phox over-expression and mitochondrial dynamics.

### p47Phox-Mediated CDK5 Activation in PV Cell Following SE

**FIGURE 4:**
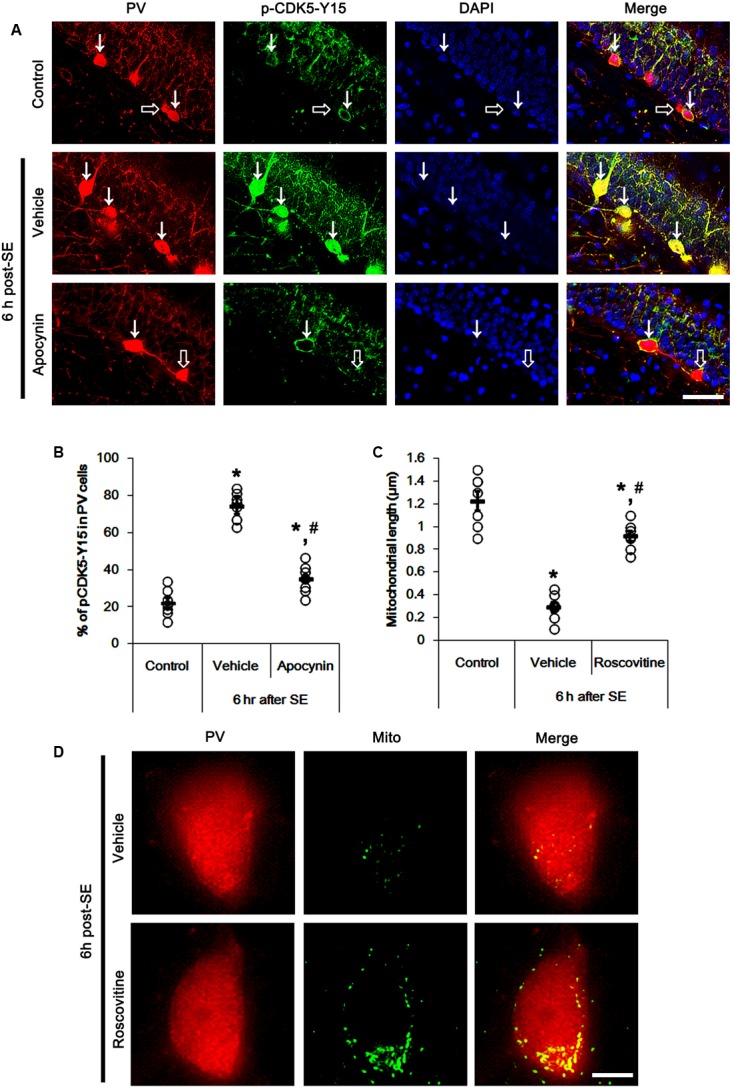
Effect of apocynin on CDK5-mediated mitochondrial fission in PV cells induced by SE. **(A)** Representative photos demonstrating CDK5-Y15 phosphorylation in PV cells. As compared to control, up-regulated CDK5-Y15 phosphorylation is observed in PV cells 6 h after SE. Apocynin attenuates SE-induced CDK5-Y15 phosphorylation in PV cells. Arrows indicate PV cells showing up-regulated CDK5-Y15 phosphorylation. Open arrows indicate PV cells showing the absence of CDK5-Y15 phosphorylation. Bar = 25 μm. **(B)** Quantification of the fraction of CDK5-Y15 phosphorylation in PV neurons. Open circles indicate each individual value. Horizontal bars indicate mean value. Error bars indicate SEM (*^∗^,^#^p* < 0.05 vs. control and vehicle, respectively; *n* = 7, respectively). **(C)** Quantification the effect of roscovitine on mitochondrial length. Open circles indicate each individual value. Horizontal bars indicate mean value. Error bars indicate SEM (*^∗^,^#^p* < 0.05 vs. control and vehicle, respectively; *n* = 7, respectively). **(D)** Representative photos demonstrating the effect of roscovitine on mitochondrial fission in PV cells induced by SE. As compared to vehicle, roscovitine attenuates SE-induced mitochondrial fragmentation. Bar = 5 μm.

**FIGURE 5:**
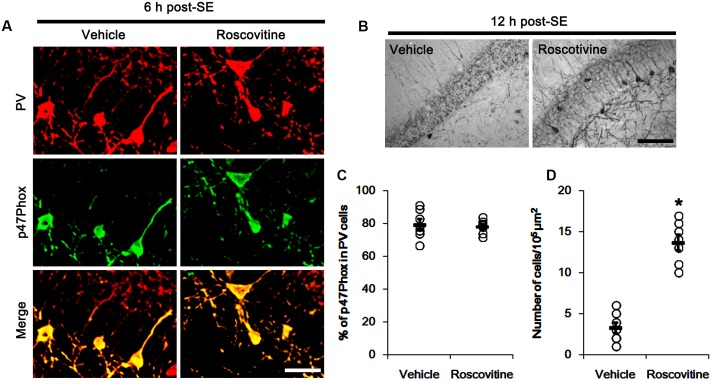
Effect of roscovitine on p47Phox expression and PV cell loss induced by SE. **(A)** Representative photos demonstrating p47Phox expression in PV cells following SE. Apocynin does not affect SE-induced up-regulation of p47Phox expression. Bar = 25 μm. **(B)** Representative photos demonstrating PV cells in the dentate gyrus. Apocynin attenuates SE-induced PV cell loss. Bar = 50 μm. **(C)** Quantification the effect of roscovitine on the fraction of p47Phox in PV neurons. Open circles indicate each individual value. Horizontal bars indicate mean value. Error bars indicate SEM (*n* = 7, respectively). **(D)** Quantification the effect of roscovitine on PV cell loss induced by SE. Open circles indicate each individual value. Horizontal bars indicate mean value. Error bars indicate SEM (*^∗^p* < 0.05 vs. vehicle; *n* = 7, respectively).

It is well known that elevated ROS and Ca^2+^ levels occur CDK5 activation, which induces further ROS accumulation in neuronal cells (Sun et al., 2008). In the present study, 19.6% of PV cells contained CDK5-Y15 phosphorylation in control animals (**Figures 4A,B**). Six hour after SE, the phosphorylation of CDK5-tyrosine (Y) 15 site was significantly elevated only in PV cells. Thus, 81.7% of PV cells contained CDK5-Y15 phosphorylation (*p* < 0.05 vs. control, **Figures 4A,B**). Apocynin effectively ameliorated SE-induced CDK5-Y15 phosphorylation in PV cells. Thus, 41.9% of PV cells showed elevated CDK5-Y15 phosphorylation (*p* < 0.05 vs. vehicle, **Figures 4A,B**). Roscovitine (a CDK5 inhibitor) prevented the excessive mitochondrial fission in PV cells induced by SE, as compared to vehicle (*p* < 0.05 vs. control, **Figures 4C,D**). Furthermore, roscovitine attenuated SE-induced PV cell loss without altered p47Phox expression in PV cells (*p* < 0.05 vs. vehicle, **Figures 5A–D**). Thus, our findings indicate that p47Phox may promote excessive mitochondrial fragmentation in PV cells by CDK5 activation.

### p47Phox-Mediated DRP1-S616 Phosphorylation in PV Cell Following SE

**FIGURE 6:**
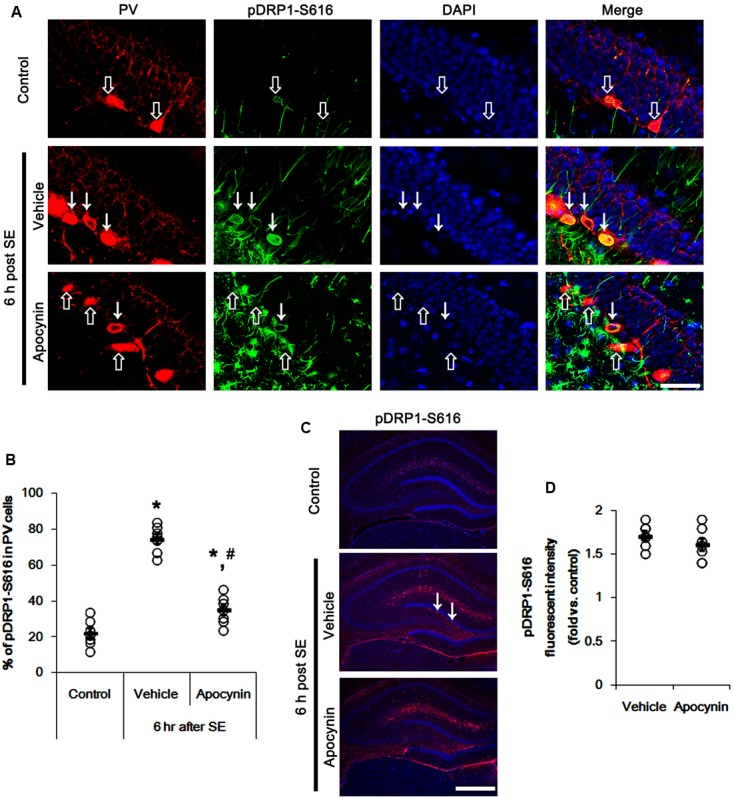
Effect of apocynin on DRP1-S616 phosphorylation in PV cells following SE. **(A)** Representative photos demonstrating DRP1-S616 phosphorylation in PV cells. As compared to control, up-regulated DRP1-S616 phosphorylation is observed in PV cells 6 h after SE. Apocynin attenuates SE-induced DRP1-S616 phosphorylation in PV cells. Arrows indicate PV cells showing up-regulated DRP1-S616 phosphorylation. Open arrows indicate PV cells showing the absence of DRP1-S616 phosphorylation. Bar = 25 μm. **(B)** Quantification of the fraction of DRP1-S616 phosphorylation in PV neurons. Open circles indicate each individual value. Horizontal bars indicate mean value. Error bars indicate SEM (*^∗^,^#^p* < 0.05 vs. control and vehicle, respectively; *n* = 7, respectively). **(C)** Low magnification photos demonstrating DRP1-S616 phosphorylation in the hippocampus. As compared to control, up-regulated DRP1-S616 phosphorylation is observed in subgranular neurons (arrows) as well as non-neuronal cells 6 h after SE. Apocynin attenuates SE-induced DRP1-S616 phosphorylation in subgranular neurons, but not in non-neuronal cells. Bar = 400 μm. **(D)** Quantification of the pDRP1-S616 fluorescent intensity in non-neuronal cells. Open circles indicate each individual value. Horizontal bars indicate mean value. Error bars indicate SEM (*n* = 7, respectively).

**FIGURE 7:**
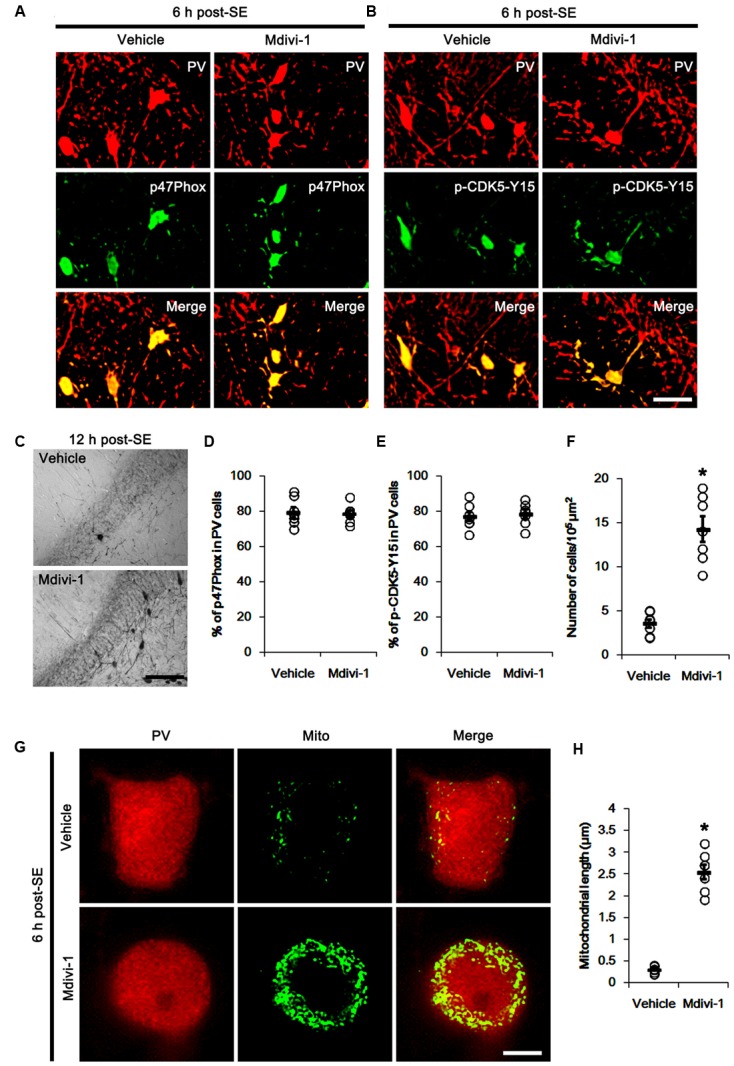
Effect of Mdivi-1 on p47Phox expression, CDK5 phosphorylaiton and PV cell loss induced by SE. **(A,B)** Representative photos demonstrating the effect of Mdivi-1 on p47Phox expression and CDK5 phosphorylation in PV cells following SE. Mdivi-1 does not affect SE-induced up-regulations of p47Phox expression and CDK5 phosphorylaiton. Bar = 25 μm. **(C)** Representative photos demonstrating PV cells in the dentate gyrus. Mdivi-1 attenuates SE-induced PV cell loss. Bar = 50 μm. **(D)** Quantification the effect of Mdivi-1 on the fraction of p47Phox in PV neurons. Open circles indicate each individual value. Horizontal bars indicate mean value. Error bars indicate SEM (*n* = 7, respectively). **(E)** Quantification the effect of Mdivi-1 on the fraction of CDK5-Y15 phosphoryaltion in PV neurons. Open circles indicate each individual value. Horizontal bars indicate mean value. Error bars indicate SEM (*n* = 7, respectively). **(F)** Quantification the effect of Mdivi-1 on PV cell loss induced by SE. Open circles indicate each individual value. Horizontal bars indicate mean value. Error bars indicate SEM (*^∗^p* < 0.05 vs. vehicle; *n* = 7, respectively). **(G)** Representative photos demonstrating the effect of Mdivi-1 on mitochondrial fission in PV cells induced by SE. As compared to vehicle, Mdivi-1 attenuates SE-induced mitochondrial fragmentation. Bar = 5 μm. **(H)** Quantification the effect of Mdivi-1 on mitochondrial length. Open circles indicate each individual value. Horizontal bars indicate mean value. Error bars indicate SEM (*^∗^p* < 0.05 vs. vehicle; *n* = 7, respectively).

The remaining question is how p47Phox-mediated CDK5 activation facilitated mitochondrial fragmentation in PV cells following SE. Mitochondrial dynamics are regulated by various molecules, such as mitofusin 1/2 (MFN1/2), optic atrophy 1 (OPA1) and dynamin-related proteins 1 (DRP1; Chan, 2006). Among them, DRP1 is a key molecule for mitochondrial fission (Smirnova et al., 2001). DRP1 activity is differently regulated by its phosphorylation: Serine (S) 616 site phosphorylation facilitates mitochondrial fission, but S637 site phosphorylation inhibits it (Kashatus et al., 2011). Since CDK5 triggers mitochondrial fission by increased DRP1-S616 phosphorylation (Xie et al., 2015), it is likely that p47Phox-mediated CDK5 activation may evoke excessive mitochondrial fragmentation in PV cells via DRP1-S616 phosphorylation. To confirm this hypothesis, we validated whether p47Phox activity enhances DRP1 phosphorylation in PV cells. In control animals, 22.3% of PV cells contained DRP1-S616 phosphorylation (**Figures 6A,B**). Six h after SE, 74.7% of PV cells showed up-regulation of DRP1-S616 phosphorylation (*p* < 0.05 vs. control, **Figures 6A,B**). Consistent with our previous study (Ko et al., 2016), DRP1-S616 phosphorylation level was also increased in astrocytes. Apocynin effectively attenuated SE-induced DRP1-S616 phosphorylation in PV cells. Thus, 35.1% of PV cells showed enhanced DRP1-S616 phosphorylation (*p* < 0.05 vs. vehicle, **Figures 6A,B**). However, apocynin did not affect DRP1-S616 phosphorylation in astrocytes (**Figures 6C,D**). To address the issue of whether an excessive mitochondrial fission leads to PV cell loss in response to SE, furthermore, we applied Mdivi-1 (a DRP1 inhibitor). Mdivi-1 effectively prevented mitochondrial fragmentation in PV cells and the loss of PV neurons induced by SE (*p* < 0.05 vs. vehicle), although it did not affect p47Phox expression and p-CDK5-Y15 phosphorylation (**Figures 7A–H**). Taken together, these findings indicate that up-regulated p47Phox expression/activity may result in mitochondrial fragmentation in PV cells by enhance DRP1-S616 phosphorylation following SE.

## Discussion

**FIGURE 8:**
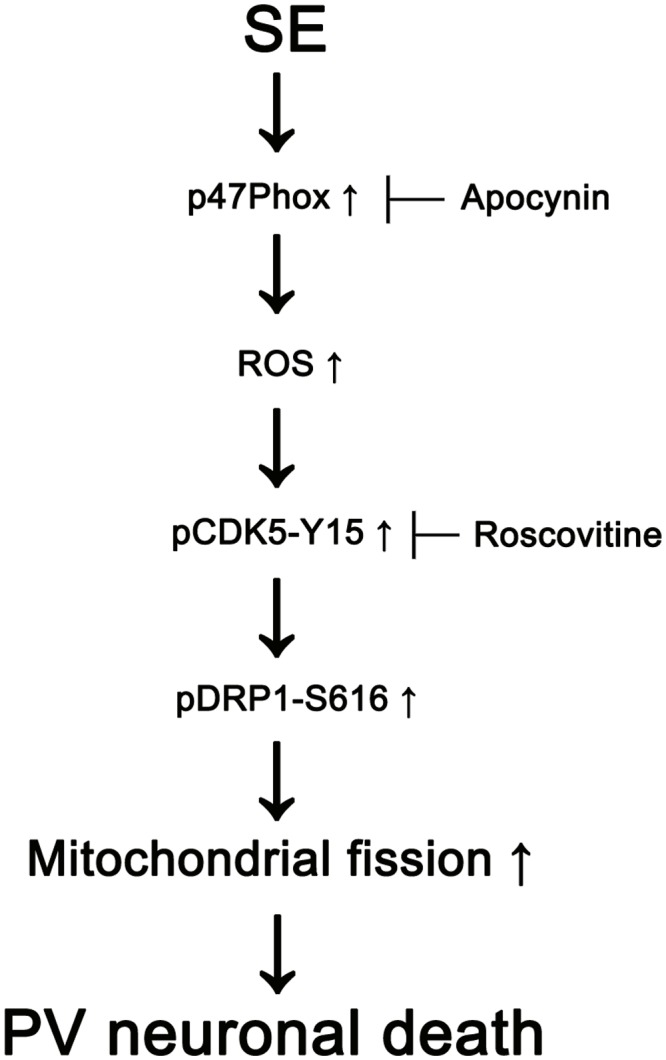
Scheme of role of p47Phox in PV cell loss induced by SE. SE increases p47Phox activity in PV cells. Subsequently, p47Phox increases ROS synthesis. ROS-mediated CDK5 activation phosphorylates DRP1 at S616 site, which leads to PV cell loss due to excessive mithocondrial fission.

The major findings of the present study are that transient up-regulation of p47Phox expression responses to pilocarpine-induced SE evoked the rapid PV cell loss via excessive CDK5/DRP1-mediated mitochondrial fission (**Figure 8**).

The loss of PV cells in the hilus of the dentate gyrus is one of the most rapid and dramatic events induced by SE. The PV cell loss is already observed in the acute phase (1 day-post SE) and further worsened to less than 10% of control values 2 months after SE, which results in the decrease in GABA outflow (Soukupová et al., 2014). Since PV is responsible for the fast-spiking capability of GABAergic neurons, PV cells play an important role in the responsiveness of fast-GABAergic inhibitory transmission with a rapid adaptation in response to repetitive stimuli. Thus, the PV cell loss allows the development of uncontrolled discharges and one of important causes of epileptogenic processes (Sloviter, 1991; Sloviter et al., 2003). However, the molecular mechanisms render PV cells more vulnerable to SE are still unclear. In the present study, we found that transient up-regulation of p47Phox expression in PV cells 6 h after SE, and that apocynin alleviated SE-induced PV cell loss alongside the prevention of up-regulation and membrane translocation of p47Phox. These findings are consistent with previous studies demonstrating that seizure activity results in the translocation of p47Phox from hippocampal cytosol to membrane fractions (Patel et al., 2005), and Nox-derived ROS production triggers cognitive impairment through PV interneuron loss (Qiu et al., 2016; Zhang et al., 2016). Therefore, our findings indicate that transient p47Phox overexpression may be one of causes for rapid PV cell loss induced by SE.

Oxidative stress by ROS contributes to neuronal death by mitochondrial dysfunctions as well as other harmful reactions including DNA damage, protein/lipid peroxidation, protein aggregation and proteosome malfunction (Jahani-Asl et al., 2007; Zhu et al., 2007). Indeed, apocynin and p47Phox genetic inhibition attenuated MA-induced neuronal toxicity via preventing mitochondrial burdens (Dang et al., 2016). Mitochondria are dynamic organelles that continuously change their morphology through fusion and fission events in response to intracellular circumstances, which is closely linked to cell death mechanisms (Cheung et al., 2007; Detmer and Chan, 2007; Jahani-Asl et al., 2007; Chen and Chan, 2009; Rintoul and Reynolds, 2010). In particular, excessive mitochondrial fragmentations provoke the impaired mitochondrial function, which increases susceptibility to apoptotic stimuli (Campello and Scorrano, 2010). Furthermore, mitochondrial fission directly enables increased mitochondrial ROS production, and further deteriorates oxidative damage secondary to Nox-derived ROS (Yu et al., 2006, 2008). In the present study, SE-induced p47Phox overexpression led to aberrant mitochondrial fissions by DRP1-S616 phosphorylation in PV cells. This p47Phox overexpression was accompanied by the increased CDK5-Y15 phosphorylation. Furthermore, apocynin effectively mitigated SE-induced CDK5 phosphorylation in PV cells. CDK5 is a unique CDK that plays a role in various neuronal activities unrelated to the cell cycle events, such as DRP1-S616 phosphorylation (Lai and Ip, 2009; Xie et al., 2015). Based on the ROS-mediated CDK5 activation (Sun et al., 2008) and the present data, our findings indicate that Nox-derived ROS may increase CDK5 activity and facilitate DRP1-mediated mitochondrial fission in PV cells. Indeed, inhibition of CDK5 activity abrogates the increase in mitochondrial fission by inhibiting DRP1 activity in Huntington’s disease model (Cherubini et al., 2015) and NMDA-induced neuronal loss (Jahani-Asl et al., 2015). The present data also reveal that roscovitine ameliorated SE-induced excessive mitochondrial fission. In addition, inhibition of excessive mitochondrial fission by Mdivi-1 effectively attenuated SE-induced PV cell loss without the changed p-CDK5-Y15 phosphorylation. Therefore, our findings suggest that p47Phox-mediated CDK5 activation may be one of the essential up-stream signal pathways that lead to excessive DRP1-dependent mitochondrial fragmentation in PV cells.

On the other hand, hippocampal neurons have the heterogeneous vulnerability in response to various insults. Dentate hilar neurons and CA1-3 pyramidal cell are extremely vulnerable to insults, while dentate granule cells are resistant (Ordy et al., 1993; Mathern et al., 1995; Wittner et al., 2001; Kim et al., 2011; Ryu et al., 2011). Interestingly, hippocampal neurons also show the distinct cell death patterns in the different hippocampal regions following SE: CA1 pyramidal cell death is necrotic rather than apoptotic (Kim et al., 2014; Ko et al., 2015; Hyun et al., 2016), but PV cells is caspase-3 dependent apoptotic (Kang et al., 2006). Recently, we have reported that the impaired mitochondrial fission induces programmed necrosis in CA1 pyramidal cells following SE (Kim et al., 2014; Ko et al., 2015; Hyun et al., 2016). Unlike CA1 neurons, the present study reveals that SE resulted in PV cell loss through excessive mitochondrial fragmentation, and Mdivi-1 effectively ameliorated SE-induced PV cell loss. Similar to the case of PV cell loss in the present study, inhibition of mitochondrial fission by Mdivi-1 attenuates the SE-induced neuronal death in hippocampus via suppression of the ROS-mediated mitochondrial apoptosis pathway in 60 min-SE duration models (Qiu et al., 2013; Xie et al., 2013). However, the effects of Mdivi-1 in DRP1 expression are distinctly described: Mdivi-1 attenuates SE-induced up-regulation of DRP1 expression (Qiu et al., 2013) or not (Xie et al., 2013). Regardless of the difference of models (60 min-SE induction in previous papers and 2 h-SE duration in the present study) and the effect of Mdivi-1 on DRP1 expression, it is likely that excessive mitochondrial fission may result in apoptotic neuronal death. Therefore, our findings indicate that the underlying mechanisms of SE-induced neuronal death may be dependent on the patterns of impaired mitochondrial dynamics. Indeed, dysfunction of mitochondrial fission (aberrant mitochondrial elongation) exerts necrotic cell death via a decrease in mitochondrial bioenergetics, while excessive mitochondrial fragmentation induces apoptotic cell death (Youle and Karbowski, 2005; Parone et al., 2008; DuBoff et al., 2012; Qiu et al., 2013; Xie et al., 2013; Kim et al., 2014). Similar to neurons, SE results in the differential mitochondrial dynamics in astrocytes, which are closely relevant to the distinct astroglial responses (Ko et al., 2016). Therefore, our findings suggest that the properties of mitochondrial dynamics may be distinct from each cell subpopulation, which influences on the cell viability or cell death pattern in response to SE.

Recently, we have reported that SE enhances pDRP1-S616 level in astrocytes within molecular layer of the dentate gyrus 3 days after SE. These SE-induced DRP1-S616 phosphorylations in astrocytes are effectively attenuated by roscovitine. Thus, we have suggested that CDK5 might play an important role in DRP1-S616 phosphorylation in astrocytes following SE (Hyun et al., 2017). However, the up-stream regulators of CDK5 phosphorylation in astrocyte are still unclear. In the present study, SE also increased pDRP1-S616 level in astrocytes 6 h after SE, although CDK5-Y15 phosphorylation in astrocytes was unaltered. Furthermore, apocynin could not affect DRP1 S-616 phosphorylation in astrocytes. These findings indicated that p47Phox may activate CDK5-mediated DRP1-616 phosphorylation in PV neurons, but not in astrocytes. Taken together, it is likely that CDK5 may be one of common kinases of DRP1-S616 in PV neurons (in the early stage) and astrocytes (in the later stage) following SE, but the up-steam activators of CDK5 may be different between PV neurons (p47Phox in the present study) and astrocytes (unknown). Further studies are needed to elucidate the signaling pathway for DRP1-S616 phosphorylation in astrocytes.

## Conclusion

We propose that p47Phox/CDK5/DRP1 axis may be one of the important upstream signal pathways for excessive mitochondrial fission, which leads to PV cell degeneration following SE.

## Author Contributions

T-CK designed and supervised the project. J-EK and T-CK performed the experiments described in the manuscript and analyzed the data. J-EK and T-CK wrote the manuscript.

## Conflict of Interest Statement

The authors declare that the research was conducted in the absence of any commercial or financial relationships that could be construed as a potential conflict of interest.
