# Supplementary material for: p47Phox/CDK5/DRP1-Mediated Mitochondrial Fission Evokes PV Cell Degeneration in the Rat Dentate Gyrus Following Status Epilepticus
Source: Front Cell Neurosci. 2017 Sep 1;11:267. doi: 10.3389/fncel.2017.00267 (PMC5585136; doi:10.3389/fncel.2017.00267)
Supplement: Supplementary file 1 [file Data_Sheet_1.PDF]

Supplementary information

p47Phox/CDK5/DRP1-mediated mitochondrial fission evokes PV cell degeneration in the rat dentate gyrus following status epilepticus

Ji-Eun Kim, Tae-Cheon Kang*

Department of Anatomy and Neurobiology, Institute of Epilepsy Research, College of Medicine, Hallym University, Chuncheon 200-702, South Korea.

Running title: p47Phox/CDK5/DRP1-mediated PV cell degeneration

* Correspondence should be addressed to T-CK (e-mail: tckang@hallym.ac.kr)

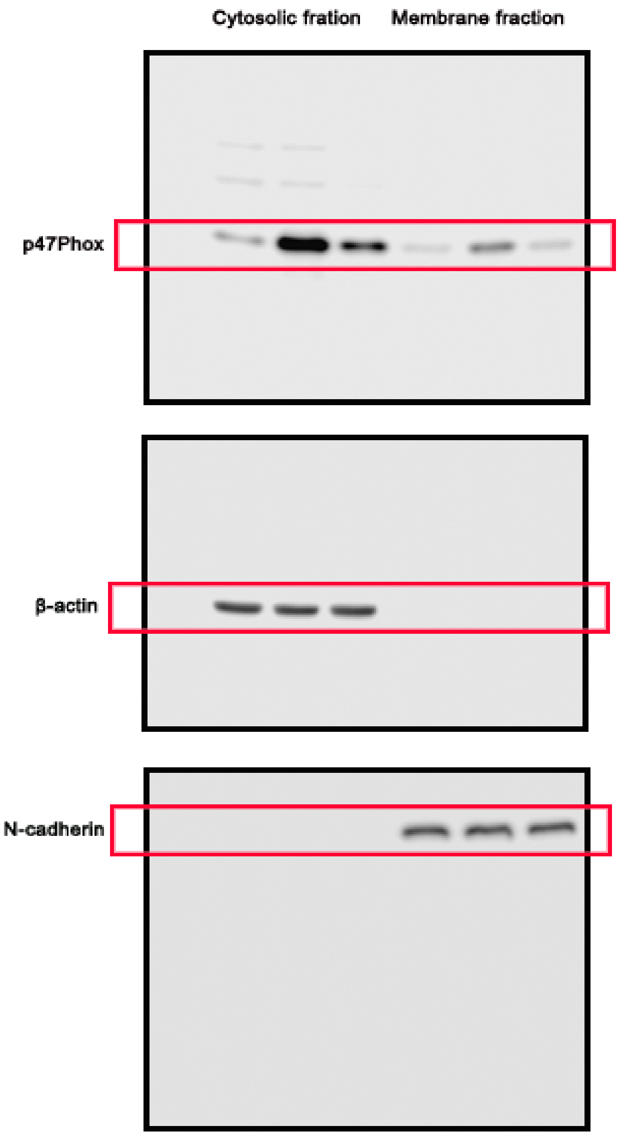

Supplementary Fig. 1. Full-length gel images of western blot data in Fig. 3. The cropped parts of western blots are indicated with boxes.
